# Supplementary material for: Diagnostic intervention improved health-related quality of life among teenagers with food allergy
Source: PLoS One. 2024 Jan 11;19(1):e0296664. doi: 10.1371/journal.pone.0296664 (PMC10783743; doi:10.1371/journal.pone.0296664)
Supplement: S1 Table — (DOCX) [file pone.0296664.s001.docx]

**ADDITIONAL FILES**

**Table S1. Scores in KIDSCREEN-52 domains before intervention among girls and boys, with and without food allergy, respectively.**

| **KIDSCREEN-52 domains** | **Girls** | |  | **Boys** | |  |
| --- | --- | --- | --- | --- | --- | --- |
| **Before** | **Food allergy** | **Non-Food allergy** |  | **Food allergy** | **Non-Food allergy** |  |
|  | **Median (Min-Max)** | **Median (Min-Max** |  | **Median (Min-Max)** | **Median (Min-Max)** |  |
|  | **Mean (SD)** | **Mean (SD)** | **p-value** | **Mean (SD)** | **Mean (SD)** | **p-value** |
| Physical well-being | 49.6 (20.7-73.2) | 49.6 (30.6-73.2) | 0.968 | 49.6 (38.5-73.2) | 49.6 (28.1-73.2) | 0.881 |
|  | 46.5 (7.8) | 46.6 (5.7) |  | 47.5 (6.6) | 46.6 (7.6) |  |
| Psychological well-being | 51.8 (31.5-68.5) | 51.8 (20.4-68.5) | 0.524 | 51.8 (36.9-68.5) | 54.5 (32.8-68.5) | 0.404 |
|  | 53.5 (9.8) | 52.1 (9.5) |  | 53.2 (8.8) | 54.6 (8.0) |  |
| Moods and emotions | 52.7 (35.7-70.9) | 54.0 (33.6-70.9) | 0.845 | 57.4 (29.0-70.9) | 57.4 (36.7-70.9) | 0.737 |
|  | 53.3 (10.0) | 53.8 (10.5) |  | 56.5 (11.1) | 57.3 (9.8) |  |
| Self perceptions | 51.0 (37.9-69.8 | 52.2 (33.2-69.8) | 0.780 | 55.4 (40.5-69.8) | 55.4 (40.5-69.8) | 0.359 |
|  | 53.0 (9.1) | 53.4 (10.4) |  | 56.6 (10.2) | 58.1 (8.2) |  |
| Autonomy | 49.7 (29.2-68.8) | 50.8 (33.7-68.8) | 0.266 | 53.2 (31.6-68.8) | 53.2 (39.0-68.8) | 0.399 |
|  | 51.7 (10.7) | 53.5 (8.9) |  | 52.5 (10.1) | 54.6 (7.9) |  |
| Parent relation and home life | 54.6 (37.0-65.9) | 54.6 (25.3-65.9) | 0.739 | 54.6 (30.2-65.9) | 54.6 (30.7-65.9) | 0.828 |
|  | 54.3 (7.6) | 54.6 (9.4) |  | 55.0 (9.6) | 55.9 (8.1) |  |
| Financial resources | 56.3 (37.5-62.9) | 56.3 (35.1-62.9) | 0.822 | 62.9 (41.9-62.9) | 56.3 (37.5-62.9) | 0.505 |
|  | 55.7 (7.9) | 56.1 (7.7) |  | 56.6 (7.8) | 55.4 (8.0) |  |
| Social support and peers | 51.3 (30.9-71.5) | 54.9 (35.4-71.5) | 0.470 | 48.4 (32.5-71.5) | 50.2 (30.9-71.5) | 0.523 |
|  | 53.3 (9.7) | 54.1 (9.1) |  | 50.6 (9.4) | 51.8 (9.3) |  |
| School environment | 56.4 (30.5-73.8) | 54.2 (14.0-73.8) | 0.855 | 54.2 (39.5-73.8) | 52.2 (33.9-73.8) | 0.688 |
|  | 56.2 (9.7 | 56.6 (11.0) |  | 54.4 (9.5) | 53.5 (8.6) |  |
| Social acceptance and bullying | 58.8 (29.1-58.9) | 58.8 (31.1-58.9) | 0.060 | 58.8 (29.1-58.9) | 58.8 (11.0-58.9) | 0.644 |
|  | 52.8 (9.0) | 55.8 (6.4) |  | 53.5 (8.2) | 51.9 (10.5) |  |
