# Supplementary material for: Diagnostic intervention improved health-related quality of life among teenagers with food allergy
Source: PLoS One. 2024 Jan 11;19(1):e0296664. doi: 10.1371/journal.pone.0296664 (PMC10783743; doi:10.1371/journal.pone.0296664)
Supplement: S2 Table — (DOCX) [file pone.0296664.s002.docx]

**ADDITIONAL FILES**

**Table S2. Scores in KIDSCREEN-52 domains after intervention among girls and boys, with and without food allergy, respectively.**

| **KIDSCREEN-52 domains** | **Girls** | |  | **Boys** | |  |
| --- | --- | --- | --- | --- | --- | --- |
| **After** | **Food allergy** | **Non-Food allergy** |  | **Food allergy** | **Non-Food allergy** |  |
|  | **Median (Min-Max)** | **Median (Min-Max** |  | **Median (Min-Max)** | **Median (Min-Max)** |  |
|  | **Mean (SD)** | **Mean (SD)** | **p value** | **Mean (SD)** | **Mean (SD)** | **p value** |
| Physical well-being | 48.4 (25.1-73.2) | 47.1 (28.1-73.2) | 0.775 | 47.1 (25.1-73.2) | 49.6 (30.6-73.2) | 0.649 |
|  | 45.4 (7.5) | 45.1 (5.8) |  | 46.8 (8.3) | 45.8 (7.1) |  |
| Psychological well-being | 51.8 (16.7-68.5) | 49.3 (28.6-68.5) | 0.440 | 51.8 (25.2-68.5) | 53.1 (25.2-68.5) | 0.901 |
|  | 50.1 (11.2) | 49.1 (9.5) |  | 51.9 (10.7) | 52.7 (8.2) |  |
| Moods and emotions | 51.3 (29.0-70.9) | 51.3 (32.5-70.9 | 0.993 | 54.0 (33.6-70.9) | 54.0 (31.4-70.9) | 0.622 |
|  | 50.7 (10.6) | 51.1 (10.6) |  | 54.1 (11.0) | 55.6 (10.1) |  |
| Self perceptions | 46.1 (25.8-69.8) | 46.1 (31.2-69.8) | 0.989 | 55.4 (40.5-69.8) | 52.2 (34.9-69.8) | 0.176 |
|  | 47.9 (10.8) | 47.9 (8.2) |  | 57.6 (9.9) | 54.5 (8.7) |  |
| Autonomy | 50.8 (37.4-68.8) | 50.8 (37.4-68.8) | 0.747 | 60.5 (33.7-68.8) | 53.2 (39.0-68.8) | 0.496 |
|  | 53.2 (9.3) | 53.2 (7.8) |  | 56.0 (11.1) | 54.1 (7.6) |  |
| Parent relation and home life | 51.8 (41.1-65.9) | 51.8 (31.6-65.9) | 0.679 | 54.6 (35.7-65.9) | 51.8 (34.3-65.9) | 0.556 |
|  | 53.7 (8.0) | 52.7 (9.2) |  | 54.5 (10.2) | 53.1 (8.4) |  |
| Financial resources | 56.3 (35.1-62.9) | 56.3 (23.2-62.9) | 0.824 | 52.4 (37.5-62.9) | 56.3 (35.1-62.9) | 0.710 |
|  | 53.7 (9.5) | 54.0 (9.6) |  | 54.1 (8.2) | 54.6 (8.6) |  |
| Social support and peers | 52.4 (9.4-71.5) | 52.4 (22.0-71.5) | 0.470 | 48.4 (9.4-71.5) | 50.2 (9.4-71.5) | 0.144 |
|  | 52.1 (11.3) | 54.0 (10.2) |  | 46.9 (11.6) | 50.8 (11.2) |  |
| School environment | 52.2 (28.4-73.8) | 52.2 (30.5-73.8) | 0.855 | 50.4 (32.3-73.8) | 52.2 (35.4-73.8) | 0.955 |
|  | 52,0 (8.4) | 53.2 (8.9) |  | 52.5 (9.7) | 52.0 (8.7) |  |
| Social acceptance and bullying | 58.8 (31.1-58.9) | 58.8 (42.2-58.9) | 0.456 | 58.8 (33.1-58.9) | 58.8 (33.1-58.9) | 0.329 |
|  | 55.7 (7.1) | 56.9 (5.0) |  | 53.8 (7.8) | 55.1 (7.4) |  |

SD: standard deviation
