# Supplementary material for: Diagnostic intervention improved health-related quality of life among teenagers with food allergy
Source: PLoS One. 2024 Jan 11;19(1):e0296664. doi: 10.1371/journal.pone.0296664 (PMC10783743; doi:10.1371/journal.pone.0296664)
Supplement: S1 File — (PDF) [file pone.0296664.s003.pdf]

Regionala
etikprövningsnämnden
i Umeå
Avdelningen för medicinsk
forskning

REGIONALA ETIKPRÖVNINGSNÄMNDEN · UMEÅ ·
EPN

PROTOKOLLSUTDRAG
sammanträdesdag
2010-09-07

Ärende, beslut eller annan åtgärd

....

Dnr
2010-247-31M

Punkt 4
Föredragande: Erik Forestier
Forskningshuvudman
Västerbottens läns landsting
Företrädare
Erik Bergström, verksamhetschef, Barn- och ungdomscentrum, NUS
Forskare
Anna Winberg, Barn- och ungdomskliniken, NUS
Projekttitel
Undersökning av förekomst av födoämnesöverkänslighet i en populationsbaserad kohort av skolbarn i norra Sverige – validering med dubbel blind placebo kontrollerade födoämnesprovokationer, inflammatoriska markörer samt dess påverkan på livskvalitet och nutritionsstatus.

Beslut
Projektet godkänns.

Nämnden har dock följande påpekanden:

1) Barnen som inbjuds till studien skall ha ett eget anpassat informationsbrev.

2) Då kvarvarande blodprov skall doneras för framtida forskning skall det vara möjligt att ge dokumenterat samtycke till detta i samtyckeshandlingen genom att tacka ja eller nej till donation.

3) I samtyckeshandlingen skrivs ”Förälderns underskrift”, detta bör ändras till ”Vårdnadshavarens”.

....

Vid protokollet
Gunnel Eriksson

Justeras
Eric Lowén
Bruno Hägglöf

Bestyrkes

Gunnel Eriksson

Kopia till
Behörig företrädare

Regionala
etikprövningsnämnden
i Umeå
Avdelningen för medicinsk
forskning

PROTOKOLLSUTDRAG
sammanträdesdag
2010-09-07

Ärende, beslut eller annan åtgärd

....

Forts.
Dnr
2010-247-31M

4) Redogörelsen för statistiska underlaget för studiepopulationens storlek (punkt 3:3) ger inte möjlighet att värdera om populationsurvalet är tillräckligt stort (eller onödigt stort) för att besvara studiens frågeställningar.

Föreslagen provhantering är förenlig med biobankslagen.

....

Vid protokollet
Gunnel Eriksson

Bestyrkes

Gunnel Eriksson

Justeras
Eric Lowén
Bruno Hägglöf

Kopia till
Behörig företrädare

Regionala etikprövningsnämnden
i Umeå
Avdelningen för medicinsk
forskning

BESLUT
2011-02-09

Anna Winberg
Barn- och ungdomskliniken
NUS
901 85 Umeå

Dnr 2011/34-32M (tillägg till dnr 2010/247-31M)
Undersökning av förekomst av födoämnesöverkänslighet i en populationsbaserad kohort av skolbarn i norra Sverige – validering med dubbel blind placebo kontrollerade födoämnesprovokationer, inflammatoriska markörer samt dess påverkan på livskvalitet och nutritionsstatus. ID: ADIOS

Projektet är tidigare godkänt.

Insänd tilläggsansökan som inkom till myndigheten 2011-01-25 **godkänns** efter granskning av de vetenskapliga sekreterarna Bruno Hägglöf och Erik Lundgren i samråd med ordföranden Anders Iacobæus.

Nämnden påpekar att uppdateringen av forskningspersonsinformationen ska skickas in till nämnden.

Anders Iacobæus, ordförande
Regionala etikprövningsnämnden i Umeå
Avdelningen för medicinsk forskning
Samverkanshuset
Universitetsområdet
901 87 Umeå

Kopia
Erik Bergström, verksamhetschef
