# Supplementary material for: Diagnostic intervention improved health-related quality of life among teenagers with food allergy
Source: PLoS One. 2024 Jan 11;19(1):e0296664. doi: 10.1371/journal.pone.0296664 (PMC10783743; doi:10.1371/journal.pone.0296664)
Supplement: S2 File — (DOCX) [file pone.0296664.s004.docx]

[English translation of ethical approval letter] PROTOCOL

Regional Ethical Committee in Umeå Day of meeting
Department of Medical Research 2010-09-07

**Matter, decision or other measure**

………………………………………………………………….

**Dnr Point 4
2010-247-31M** Person reporting on the case: Erik Forestier
 Principal organisation for the research
 Västerbotten County Council
 Representative
 Erik Bergström, head of the Pediatric Clinic, Norrland University
 Hospital (NUS)
 Researcher
 Anna Winberg, Pediatric Clinic, NUS
 Project title
 Investigation of prevalence of food hypersensitivity in a population-
 based cohort of school children in northern Sweden – validation by
 double blind placebo controlled food provocations, inflammatory
 markers and their impact on quality of life and nutritional status.

**Decision**
 The project is approved

The committee has the following notes:

1. The children invited to the study should receive an individually customized information letter.
2. As the remaining blood samples are to be donated to future research it should be possible to give written consent to this in the act of consent by either agreeing or declining donation.
3. In the act of consent it reads “parent’s signature”, this should be changed to “caregiver’s signature”.
4. The declaration of the statistical basis for the sample size (point 3:3) does not allow an evaluation of whether the sample size is large enough (or unnecessarily too large) to answer the research questions.

……………………………………………………………………………...

**Minutes by** **Approval of minutes**
Gunnel Eriksson Eric Lowén
 Bruno Hägglöf

**Confirmation** Copy to
Gunnel Eriksson Representative

Regional Ethical Committee in Umeå Decision
Department of Medical Research 2011-02-09

Anna Winberg
 Pediatric Clinic
 NUS
 901 85 Umeå

**Dnr 2011/34-32M (amendment to Dnr 2010/247-31M)**

Investigation of prevalence of food hypersensitivity in a population-based cohort of school children in northern Sweden – validation by double blind placebo controlled food provocations, inflammatory markers and their impact on quality of life and nutritional status. ID: ADIOS

Project was approved previously.

The committee was sent an amendment on 2011-01-25 which is approved after vetting by the scientific secretaries Bruno Hägglöf and Erik Lundgren together with the chairman of the committee Anders Iacobaeus.

The committee notes that the update of the information letter to the participants should be sent to the committee.

Anders Iacobaeus, chairman
Regional Ethical Committee in Umeå
Department of Medical Research
Samverkanshuset
Umeå University
901 87 Umeå

Copy
Erik Bergström, head of clinic
